# Current state and future perspectives of cytochrome P450 enzymes for C–H and C=C oxygenation

**DOI:** 10.1016/j.synbio.2022.04.009

**Published:** 2022-05-08

**Authors:** Yu Yan, Jing Wu, Guipeng Hu, Cong Gao, Liang Guo, Xiulai Chen, Liming Liu, Wei Song

**Affiliations:** a State Key Laboratory of Food Science and Technology, Jiangnan University, Wuxi, 214122, China; b School of Life Sciences and Health Engineering, Jiangnan University, Wuxi, 214122, China

**Keywords:** Biocatalysis, P450 enzymes, C–H and C=C oxygenation

## Abstract

Cytochrome P450 enzymes (CYPs) catalyze a series of C–H and C=C oxygenation reactions, including hydroxylation, epoxidation, and ketonization. They are attractive biocatalysts because of their ability to selectively introduce oxygen into inert molecules under mild conditions. This review provides a comprehensive overview of the C–H and C=C oxygenation reactions catalyzed by CYPs and the various strategies for achieving higher selectivity and enzymatic activity. Furthermore, we discuss the application of C–H and C=C oxygenation catalyzed by CYPs to obtain the desired chemicals or pharmaceutical intermediates in practical production. The rapid development of protein engineering for CYPs provides excellent biocatalysts for selective C–H and C=C oxygenation reactions, thereby promoting the development of environmentally friendly and sustainable production processes.

## Graphical abstract

**Figure:**
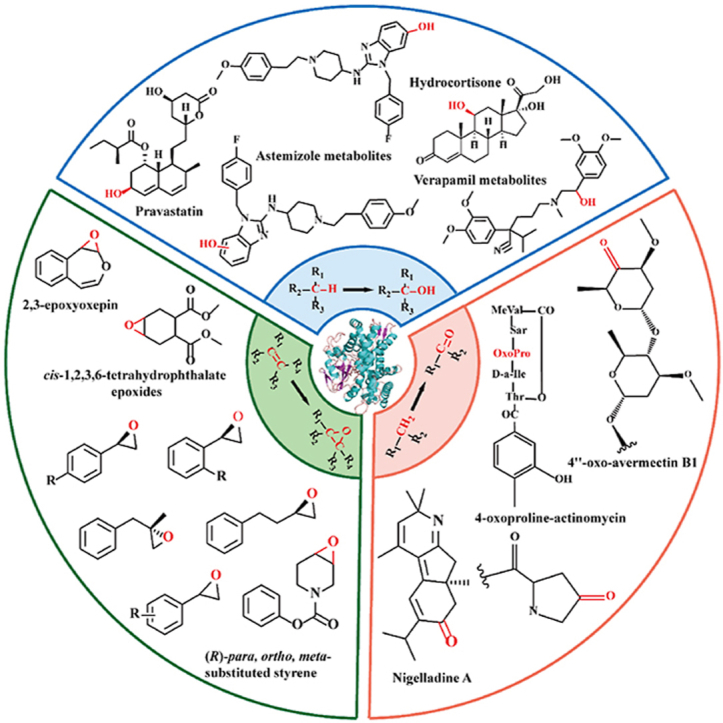

## 1 Background

Direct C–H and C=C oxygenation reactions that avoid the pre-functionalization step lead to the formation of carbon-oxygen bonds and are indispensable in synthesizing pharmaceutical intermediates and chemicals [1,2]. As carbon is more stable and common in complex compounds, its oxygenation reactions have become a research hotspot. Industrial-scale C–H and C=C oxygenation is achieved through the Hock process [3], Dakin oxidation [4] cooperated with Vilsmeier–Haack formylation [5], and various transition metal-catalyzed reactions [6], including C–O bond formation promoted by palladium [7], Sandmeyer hydroxylation [8], and Chan–Lam couplings [9]. Most C–H and C=C oxygenation reactions are performed on molecules with specific functional groups that can be transformed into -oxy substituents, such as aryl halides [10] and aryl thianthrenium salts [3]. However, how substrate molecules without special functional groups efficiently achieve selective C–H and C=C oxygenation reactions is a crucial problem in producing high value-added chemicals.

As a class of multifunctional metalloenzymes, cytochrome P450 enzymes (CYPs) have attracted recent attention. They have been extensively studied owing to their excellent catalytic capacity, various reaction types, and broad substrate range. CYPs catalyze a wide range of reactions, such as N/S-oxidation, N/O/S-dealkylation, C–C bond cleavage, epoxidation of C=C bonds, hydroxylation, and ketonization of C–H bonds, thereby having excellent application potential for medicine, food processing, chemistry, and other fields [11,12]. Unlike most oxidases, CYPs can catalyze the hydroxylation of saturated alkanes without requiring any special groups and active elements and quickly activate inert molecules by using O_2_ or H_2_O_2_, which has high economic significance for chemical production based on simple raw materials [13,14]. Furthermore, the epoxidation of carbon-carbon unsaturated bonds is a typical C=C oxygenation reaction catalyzed by CYPs, which not only accomplishes the insertion of oxygen atoms but also achieves ring formation by overcoming tension and other factors [15,16]. Surprisingly, a few CYPs can further catalyze the ketonization of hydroxyl products to eventually form C=O bonds [17,18]. However, there remain some issues to be addressed in terms of C–H and C=C oxygenation reactions by CYPs, such as how to achieve highly selective hydroxylation, improve the conversion efficiency of epoxidation, and develop more CYPs to achieve ketonization. Using DNA recombination and protein engineering, the conversion rate, regioselectivity, and enantioselectivity of C–H and C=C oxygenation reactions catalyzed by CYPs can be significantly improved, which makes CYPs highly desirable for chemical synthesis.

In this review, we outline the recent advances and related applications in three types of C–H and C=C oxygenation reactions catalyzed by CYPs: hydroxylation, epoxidation, and ketonization. Based on published reviews, we further classify and summarize the strategies to improve the catalytic efficiency and selectivity of the above mentioned reactions, thus providing a reference for protein engineering approaches to specific problems. In addition, we summarize the synthetic applications of C–H and C=C oxygenation reactions catalyzed by CYPs and prospect the development of new functions of CYPs based on the latest research findings. Overall, this review comprehensively summarizes the current research and application of C–H and C=C oxygenation reactions catalyzed by CYPs and emphasizes the potential of CYPs. Undoubtedly, the P450 enzyme functional system provides a template and new insights for large-scale C–H and C=C oxygenation reactions.

### 1.1 Hydroxylation catalyzed by CYPs

Hydroxylation of the C–H bond is the most common catalytic reaction for CYPs, which rapidly activates inert molecules by introducing OH groups. However, the hydroxylation of alkene substrates generally has a low selectivity or an inability to introduce an oxygen atom into the desired position. In this section, taking representative aliphatic alkanes as examples, we describe the common issues, focusing on the relevant universal strategies to improve the selectivity of CYP-catalyzed hydroxylation reactions. Depending on the source of the oxygen atom, hydroxylation reactions can be classified as O_2_- or H_2_O_2_-mediated [19,20].

#### 1.1.1 O_2_-mediated hydroxylation

**Fig. 1:**
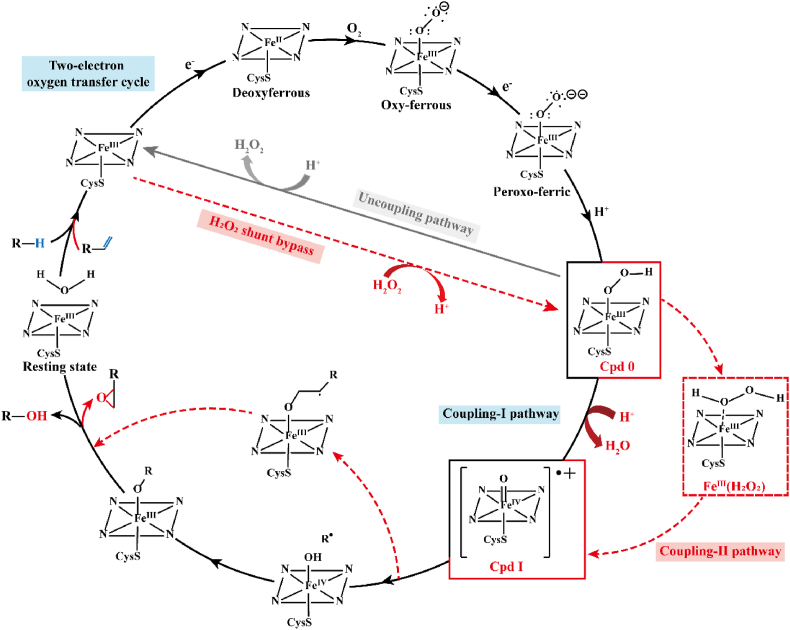
Catalytic mechanism of CYPs.

The classic O_2_-mediated two-electron oxygen transfer cycle is the main pathway of hydroxylation by CYPs. It begins with reducing the aqua-ferric resting state by accepting one electron owing to the entry of substrate molecules into the active site (Fig. 1) [21]. Subsequently, O_2_ uptake leads to the ferrous state and reductive activation generating an oxyferrous intermediate. The addition of a second electron to the oxyferrous intermediate yields a peroxo-ferric derivative and forms a hydroperoxo-ferric intermediate (Cpd 0) via distal oxygen protonation [22]. Afterwards, the second proton is transferred to the distal OH group of Cpd 0, leading to O–O bond heterolysis and water release and the formation of an electrophilic π-cation radical oxoferryl state (Cpd I) [23,24]. The above mentioned conversion of Cpd 0 to Cpd I is referred to as the coupling-I pathway. Subsequently, Cpd I oxidizes the substrate by abstracting an H atom, which decides the placement of the OH group and regioselectivity, thereby generating the caged substrate radical coordinated to the iron hydroxo complex (Cpd 2) [23,25]. Finally, the substrate is replaced by a water ligand and recombines with the protonated oxygen bound to iron, thereby forming hydroxyl products.

The catalytic mechanism of hydroxylation and epoxidation catalyzed by CYPs is shown. Black lines represent the classic O_2_-mediated two-electron oxygen transfer cycle, red dashed lines represent the hydrogen peroxide shunt bypass, gray lines represent the uncoupling pathway, and outer red dashed circles represent the involved intermediates in epoxidation different from those in hydroxylation.

According to the above mechanism, O_2_-mediated regioselectivity is mainly controlled by the position of the H-atom extracted by Cpd I. However, for benzene, steroids, or chain fatty acids, multiple C–H bonds with the same extraction probability reduce hydroxylation regioselectivity. Specific C–H bonds can increase the possibility of H-atom extraction by shortening the distance from the active site, thus improving the regioselectivity of the specific position. This is usually achieved by adjusting the substrate-binding conformation [26,27].

The substrate-binding conformation at the active site is usually adjusted by systematically enlarging the substrate pocket, introducing new binding sites, and employing scanning chimeragenesis. A larger substrate pocket provides more space for substrate molecules to adjust the binding conformation, thus allowing specific C–H bonds to be closer to the heme and affecting regioselectivity and enantioselectivity [28]. This adjustment can be achieved by replacing the active site residues with smaller ones [29]. For example, Phe87 of CYP102A1 mutated into Ala turned the substrate terminal and the *pro-S* side of the C–H bond toward the heme, leading to a 24% increase in regioselectivity at position *ω*-5 [30]. Similarly, the L354I mutant in CYP153A33 provided approximately a 76% increase in *ω*-1 selectivity[31]. The V78A and I263G mutants in CYP102A1 and the G307A and S233G mutants in CYP153A33 enhanced regioselectivity for new positions that could not be catalyzed by the wild type [[30], [32]]. In addition, introducing new binding sites can change the rigid binding conformation, which addresses the limitation of intrinsic binding anchors and acquires new polar anchors to attract different substrate groups closer to the heme [26]. For instance, the S72Y mutant in CYP102A1 increased the selectivity for position *ω*-9 by 9%28 whereas the M179Q mutant in CYP107Z14 increased the ratio of CsA-9-OH (*γ*-hydroxy-*N*-methyl-L-Leu^9^ -CsA) to CsA-4-OH up to 60%, compared with the wild-type (31%) [33]. The last strategy is the scanning chimeragenesis method [34]. By scanning the active site of highly selective CYPs, the residues near the binding substrate are transferred to the substrate recognition sites of other CYPs to form protein chimeras, thus improving their selectivity for specific substrates [35]. However, it is not widely used to adjust the substrate-binding conformations as it is often accompanied by damage to hydroxylation capacity [34]. The most representative example of scanning chimeragenesis is the fusion of CYP102A1 chimeras with the corresponding residues from CYP4C7, which showed 27% regioselectivity for position *ω*-6, where the wild-type showed difficulties in catalysis [36]. The details of the above examples were listed in Table 1.

**Table 1:** O_2_-mediated hydroxylation catalyzed by CYPs.

Substrate	Variant	Conc. (mM)	Con. (%)	Enantiomeric excess (%)	References
Octanoic acid	CYP153A33	1	–	–	[31]
	CYP153A33 G307A variant	1	20.3	98.4(*ω*)	[31]
Nonanoic acid	CYP153A33	1	1.7	97.5(*ω*), 2.5(*ω*-1)	[31]
	CYP153A33 L354I variant	1	2	24.4(*ω*), 75.6(*ω*-1)	[31]
	CYP153A33 L354F variant	1	1.2	83 (*ω*), 17(*ω*-1)	[31]
	CYP153A33 G307A variant	1	26	98.9(*ω*), 1.1(*ω*-1)	[31]
Dodecanoic acid	CYP102A1	0.5	34	49(*ω*-1), 30(*ω*-2), 21(*ω*-3)	[37]
	CYP102A1 T268A variant	0.5	10	47(*ω*-1), 34(*ω*-2), 19(*ω*-3)	[37]
	CYP102A1 F87G variant	0.5	n.r.	19(*ω*-4), 34(*ω*-5)	[28]
	CYP102A1 F87V variant	0.5	n.r.	4(*ω*-4), 3(*ω*-5)	[28]
	CYP102A1 F87S variant	0.5	n.r.	16(*ω*-4), 7(*ω*-5)	[28]
	CYP102A1 F87A/V78A/I263G variant	0.5	54	8(*ω*-4), 23(*ω*-5), 4(*ω*-6), 14(*ω*-7), 2(*ω*-8), 3(*ω*-9)	[28]
	CYP102A1 F87A/S72Y/V78A variant	0.5	71	1(*ω*-4), 3(*ω*-5), 25(*ω*-6), 16(*ω*-7), 5(*ω*-8), 9(*ω*-9)	[28]
	CYP102A1-CYP4C7 chimera: 73–78	0.25	n.r.	26(*ω*-4), 9(*ω*-5), 27(*ω*-6)	[35]
	CYP153A33	0.2	64	97(*ω*)	[31]
	CYP153A33 G307A variant -CYP102A1 fusion protein	50	12	>95(*ω*)	[38]
	CYP153A33	0.28	25.6	*ω*-specific	[39]
	CYP153A33 P136A variant	0.23	60.9	*ω*-specific	[39]
	CYP102 Krac_9955	1	nr	6(*ω*-1), 16(*ω*-2), 72(*ω*-3)	[40]
Tridecanoic acid	CYP102A1	2	nr	19(*ω*-1), 64(*ω*-2), 17(*ω*-3)	[[37], [41]]
Tetradecanoic acid	CYP153A33	1	48.4	97.1(*ω*)	[31]
	CYP153A33 G307A variant	1	68.6	96.8(*ω*)	[31]
	CYP102A1	0.5	88	48(*ω*-1), 27(*ω*-2), 25(*ω*-3)	[37]
	CYP505A30	1	nr	63(*ω*-1), 28(*ω*-2), 9(*ω*-3)	[42]
Pentadecanoic acid	CYP102A1	0.5	88	36(*ω*-1), 43(*ω*-2), 21(*ω*-3)	[37]
12-Methylmyristic acid	CYP102A1	0.5	90	85(*ω*-1), 2(*ω*-2), 13(*ω*-3)	[43]
13-Methylmyristic acid	CYP102A1	0.5	76	15(*ω*-1), 83(*ω*-2), 2(*ω*-3)	[43]
Hexadecenoic acid	CYP102A1	0.5	93	23(*ω*-1), 43(*ω*-2), 34(*ω*-3)	[37]
	CYP102A1 T268A variant	0.5	21	25(*ω*-1), 42(*ω*-2), 33(*ω*-3)	[37]
	CYP102A1-CYP4C7 chimera: 73–78	0.2	n.r.	7(*ω*-4),10(*ω*-5), 27(*ω*-6)	[34]
	CYP102A1-CYP4C7 chimera: 78–82	0.2	n.r.	8(*ω*-4),4(*ω*-5), 4(*ω*-6)	[34]
14-Methylpalmitic acid	CYP102A1	0.5	87	85(*ω*-1), 2(*ω*-2), 13(*ω*-3)	[43]
15-Methylpalmitic acid	CYP102A1	0.5	96	9(*ω*-1), 89(*ω*-2), 2(*ω*-3)	[43]

In addition, the fusion of exogenous redox proteins to improve the enzymatic efficiency of hydroxylation, combined with adjustment of the substrate-binding conformation to regulate regioselectivity, is a common combination strategy for O_2_-mediated hydroxylation with high efficiency and selectivity [44]. A representative example is the CYP105AS1 from *Amycolatopsis orientalis* fused to the RhF reductase of CYP116B1, which initially catalyzed the efficient hydroxylation of compactin to 6-epi-pravastatin. This fusion protein was further evolved to the P450(Prava) mutant to produce the pharmacologically effective pravastatin via changing the compactin binding modes and inverting its natural stereoselectivity [45]. The fusion of the diflavin reductase domain of CYP102A1 and the G307A mutant in CYP153A33 increased the conversion rate of C12 saturated fatty acids by 12%, and the yield was enhanced when the corresponding methyl ester was used as the substrate [31]. In fact, fusing exogenous redox proteins improves the catalytic efficiency of the whole cycle by reinforcing electron transport efficiency. The inefficient coupling of the electron transport system (Table 2) to P450 enzymes often becomes the rate-limiting step of the entire reaction [46]. This fusion can be achieved by linking the P450 enzyme domain and the redox system into a chimera using DNA recombination technology through simple residues (linker), thereby forming a polypeptide protein to address inefficient coupling [47,48]. For example, the RhFRED reductase domain of P450RhF from *Rhodococcus* sp was fused to P450Pikc to form a novel self-sufficient chimera with an efficient electron transport system, resulting in a fourfold increase in hydroxylation activity for both YC-17 and narbomycin [49,50]. Furthermore, the length, hydrophobicity, and the secondary structure of the linker affect the expression, coupling efficiency, and correct folding of the fusion protein. For example, in the construction of the fusion proteins of CinA and CinC of P450cin, co-expression activity was the highest when the length of the linker was 10 residues [51]. CYP102A1 reductase (BMR) was fused to the *N*-terminally modified P450 3A4 via a glycine hinge to construct the self-sufficient chimeric 3A4-BMR. Compared with 3A4-3GLY-BMR, 3A4-5GLY-BMR demonstrated a 1.26-fold increase in coupling efficiency of testosterone hydroxylation [52].

**Table 2:** **Electron transport system of CYPs** [53,54].

Classification	Electron transport chain	Redox partners	Sources
Class I	NADH → FdR → Fdx → heme	Ferredoxin reductase (FdR, FAD), Ferredoxin (Fdx, [2Fe–2S] cluster)	*Bacterial* or *Mitochondrial*
Class II	NADPH → CPR → heme	Cytochrome P450 reductase (CPR), consisting of FMN and FAD	*Bacterial* or *Mitochondrial*
Class III	NAD(P)H → FdR → Fld → heme	Ferredoxin reductase (FdR, FAD), FMN-containing flavodoxin (Fld)	*Bacterial*
Class IV	Pyruvate/CoA → OFOR → Fdx → heme	2-Oxoacid: ferredoxin oxidoreductase (OFOR)	*Bacterial*
Class V	NADPH → FdR → Fdx-heme	Ferredoxin reductase (FdR, FAD), Ferredoxin (Fdx)	*Bacterial*
Class VI	NAD(P)H → FdR → Fld-heme	Ferredoxin reductase (FdR, FAD), FMN-containing flavodoxin (Fld)	*Bacterial*
Class VII	NAD(P)H → PFOR-heme	Phthalate-family oxygenase reductase (PFOR), consisting of FMN and Fdx ([2Fe–2S] cluster)	*Bacterial*
Class VIII	NAD(P)H → BMP-heme	BMP consisting of ferredoxin reductase (FdR, FAD) and FMN	*Bacterial*
Class IX	NADH → heme	–	*Bacterial*
Class X	HEME	–	*Plant* or *Mammal*

#### 1.1.2 H_2_O_2_-mediated hydroxylation

Unlike O_2_-mediated hydroxylation, H_2_O_2_-mediated hydroxylation takes hydrogen peroxide as the sole oxygen atom source and electron donor without complex electron transport systems; therefore, only the heme domain is required [54]. Only a few peroxygenases can catalyze H_2_O_2_-mediated hydroxylation, do not require cofactors and redox protein partners, and generally show high regioselectivity or enantioselectivity [55]. Peroxygenases can directly convert the resting state into Cpd 0 by virtue of electrons contributed by H_2_O_2_, avoiding the complicated electron transport process [19]. Subsequently, H_2_O_2_ provides a proton to the proximal oxygen of Cpd 0, resulting in the formation of Fe^III^(O_2_H_2_) intermediates as a transient second oxidant, followed by O–O homolysis and release of OH radicals [56]. Finally, the hydrogen bond network locks the hydroxyl radical and forces it to extract a proton from the iron complex, thereby generating Cpd I to hydroxylate substrates with high selectivity [57]. The Cpd 0 → Fe^III^(O_2_H_2_) → Cpd I process is referred to as the coupling-II pathway, and the abbreviated cycle using H_2_O_2_ is called hydrogen peroxide shunt bypass (Fig. 1) [23].

H_2_O_2_-mediated hydroxylation using the coupling-II pathway generally shows higher regioselectivity than the coupling-I pathway, which may also be due to the strict substrate-binding pocket of peroxygenases [56]. For example, CYP152B1 catalyzes the production of (*S*)-2-hydroxymyristic acid from the natural substrate myristic acid with 94% enantiomeric excess (ee) [58]. However, hydroxylation mediated by H_2_O_2_ is often accompanied by oxidative decarboxylation (Table 3), so the regulation of reaction selectivity cannot be ignored. Thus far, the mechanism of oxidative decarboxylation as a side reaction catalyzed by CYPs remains controversial. The most convincing explanation is that oxygen rebound causes substrate hydroxylation or that a carbocation triggers decarboxylation after a hydrogen atom is extracted from the *α*- or *β*-position of the substrate to obtain the corresponding carbon radical [59,60].

**Table 3:** Two reactions catalyzed by CYP152 peroxygenases (C12 fatty acids) [19].

Substrate^a^	Enzyme	Product distribution (%)		
		Hydroxylation	Decarboxylation	Undecanal formation^b^
Dodecanoic acid	CYP152A1	72.5%	12.5%	15%
	CYP152B1	94.6%	n.r.	5.4%
	CYP152L1	27.1%	63.6%	9.3%
*cis*-2-dodecenoic acid	CYP152A1	3.2%^c^	n.r.	56.5%
	CYP152B1	n.r.^c^	n.r.	63.2%
	CYP152L1	89.3%^c^	n.r.	1.8%
*trans*-2-dodecenoic acid	CYP152A1	3.3%^c^	n.r.	58.2%
	CYP152B1	n.r.^c^	n.r.	62.4%
	CYP152L1	98.5%	n.r.	1.5%

a Reactions conditions: 0.5 mM substrate; 0.001 mM CYP152 enzyme; 0.5 mM alditol oxidase (AldO) and 10% glycerol for in situ generation of H_2_O_2_ as the oxygen source; 30 °C; 6 h.

b Undecanal formation is the sequential hydroxylation, isomerization, and decarboxylation.

c Other oxygenation reactions were detected, including epoxidation and ketonization.

Reaction selectivity is influenced by the specific substrate molecules with different carbon chain lengths and can be efficiently controlled by adjusting substrate binding via mutations in the conserved binding site [61]. The most common adjustments include mutations of conserved arginine as a binding anchor to the carboxyl-terminal of the substrate [62,63]. For example, when the substrate was a C10 saturated fatty acid, the R245L mutant of CYP152L1(OleT_JE_) increased the conversion rate of *α*-hydroxylation by 78% and decreased decarboxylation from 51% to 0.1%, whereas the R245E mutant completely lost hydroxylation activity. However, the R254L mutant showed significant decreases of 48% and 97% in hydroxylation activity for C12 and C14 saturated fatty acids, respectively [60]. The affinity of CYP152L1 for substrates decreases as the chain length of fatty acids increases [64]. In summary, a series of conserved arginine mutants influence the proton extraction process of the substrate by changing the binding conformation and controlling reaction selectivity.

### 1.2 Epoxidation catalyzed by CYPs

CYPs also catalyze the epoxidation of C=C bonds to form oxirane-containing chemicals. As a typical carbon-oxygen cyclization reaction, epoxidation involves non-rotatable unsaturated bonds and ring tension, unlike hydroxylation. In this section, we focus on the central questions of epoxidation, namely the relevant strategies for improvement of catalytic efficiency. According to the source of oxygen atoms, epoxidation reactions can be classified as O_2_- or H_2_O_2_-mediated epoxidation.

#### 1.2.1 O_2_-mediated epoxidation

In the presence of O_2_, epoxidation and hydroxylation are often observed simultaneously in olefin oxygenation catalyzed by CYPs, causing an increase in the cost of product purification and separation. For example, CYP102A1 showed a low selectivity for catalyzing *α*-isophorone oxygenation, where hydroxyl products and epoxides were found [65]. CYP MycG catalyzed the biosynthesis of mycinamicin II (M-II) from mycinamicin IV (M-IV), where 20.6% of M-IV underwent epoxidation to mycinamicin-I and 77.4% underwent sequential hydroxylation and epoxidation to M-II [66]. In fact, the mechanism of epoxidation was reported to generate a metal-carbon radical intermediate instead of Cpd 2 intermediate in hydroxylation (Fig. 1) [67,68].

To achieve specific O_2_-mediated epoxidation, the coupling-II pathway has been artificially constructed by interrupting the protonation process mediated by acid-alcohol pairs. Once the normal proton transfer through acid-alcohol pairs is interrupted, direct conversion from Cpd 0 to Cpd I is impeded, and the electrons transported by redox systems reduce O_2_ to H_2_O_2_, which induces the formation of the Fe^III^(O_2_H_2_) transient intermediate [23,69]. Nucleophilic attacks from the generated Cpd I intermediate to olefin results in the formation of an iron-alkoxy radical complex intermediate, and epoxides are finally released [70]. Collectively, disruption of the acid-alcohol pairs terminates the coupling-I pathway and initiates the coupling-II pathway in the classic two-electron oxygen transfer cycle.

The disruption of acid-alcohol pairs can be achieved by mutation of the conserved threonine, which participates in Cpd 0 protonation. The alanine mutation eliminates the OH group and leaves no other electrically charged groups to transfer protons. The most typical example is the T252A mutant of CYP101A1 from *Pseudomonas putida* that specifically catalyzes the epoxidation of 5-methylenylcamphor but is associated with a lost capacity for effective hydroxylation. In contrast, the wild-type mainly catalyzed camphor hydroxylation [23]. This was the earliest evidence for the existence of the Fe^III^(O_2_H_2_) intermediate; however, the catalytic activity of the T252A mutant was decreased by 80% [69]. This change in catalytic efficiency may be explained by the increase in uncoupling efficiency due to the generation of hydrogen peroxide. This can be demonstrated by a study of the epoxidation of 4-vinylbenzoic acid catalyzed by CYP199A4 and its T252A mutant. CYP199A4 T252A mutant showed a 1.6-fold increase in NADH oxidation rate but a 40% decrease in coupling efficiency, resulting in reduced epoxidation efficiency [71,72].

**Fig. 2:**
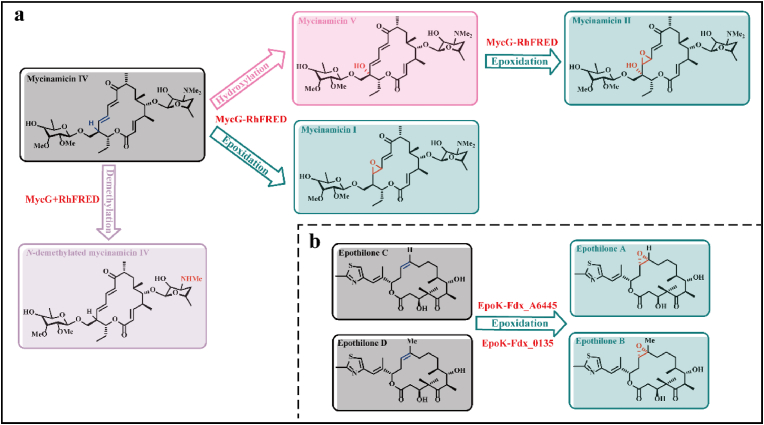
Exogenous redox proteins for O_2_-mediated epoxidation **a.** The biotransformation from mycinamicin IV catalyzed by CYP MycG with RhFRED protein; **b.** The biotransformation from epothilone C + D catalyzed by EpoK with Fdx proteins.

Owing to the presence of electron transport systems in classic cycle, the fusion of exogenous redox proteins to achieve a more efficient electron transport process is suitable for improving O_2_-mediated epoxidation activity (see section *O*_*2*_*-mediated hydroxylation* above). For example, the epoxidation of epothilone C + D catalyzed by CYP167A1 (EpoK) co-expressed with Fdx_0135, a hybrid [3Fe–4S]/[4Fe–4S] ferredoxin from *Schlegelella brevitalea* DSM 7029, had a 90.93% conversion rate, which was 1.5-fold higher than that by single EpoK; in contrast, the conversion rate of epothilone C reached 100% in whole-cell transformation where EpoK was co-expressed with Fdx_A6445 (a similar ferredoxin to Fdx_0135) (Fig. 2b) [73]. In addition to the type of exogenous proteins, the interaction between exogenous proteins and CYPs can influence enzyme activity and reaction type. For example, the CYP MycG-RhFRED fused protein showed a 2.4-fold increase in the yield of oxidized product (mycinamicins I, II, and IV) compared with CYP MycG cooperated with separated RhFRED protein; however, the new *N*-demethylation of mycinamicins IV was observed during separation (Fig. 2a) [74].

The exogenous protein has relatively less influence on self-sufficient CYPs that do not require additional redox proteins. In this case, catalytic efficiency and selectivity are mainly controlled by the substrate-binding orientation, which is influenced and regulated by residues at the active site. For instance, the CYP102A1 F87V mutant achieved 99% 14(*S*)-15(*R*)-epoxidation in arachidonic acid, whereas the wild type yielded 20% epoxidation and 80% 18(*R*)-hydroxylation [75]. For the epoxidation of 1-hexene catalyzed by CYP102A1, the SH-44 mutant had 88% (*S*)-selectivity, the RH-47 mutant had 93% (*R*)-selectivity, and the wild type had 10% (*R*)-selectivity. The epoxidation activities of the SH-44 mutant (containing F87V and I263A) and RH-47 mutant (containing I263A, A82F, and A328V) were increased by 54.5- and 30.5-fold, respectively, compared to that of wild type [76]. Substitutions in key residues closest to the heme affect the oxygenation reactions catalyzed by CYPs and the selectivity and efficiency of other reactions (for example, asymmetric amination [77] and aminohydroxylation [78]).

In particular, the modification of the heme-propionate side chains, including the introduction of hydrophobic moieties or flavin moieties, is an emerging approach to optimize the performance of heme proteins [79,80]. The heme-propionate side chains mediate the first protonation in the classic O_2_-mediated two-electron oxygen transfer cycle, facilitating the synthesis of the Cpd 0 intermediate through the hydrogen bond network of the water chain [22]. Unlike relatively matured acid-alcohol pairs strategies that regulate the second protonation, the strategies targeting the heme-propionate side chains of CYPs are still under development. However, they have shown great potential in other heme-containing enzymes. This method helped achieve optimal results in myoglobins containing heme catalytic centers. For example, introducing the benzene group as a hydrophobic substrate-binding site into propionate side chains improved the catalytic efficiency (*Kcat/K*_*M*_) of reconstituted myoglobin by 14 times [81]. Moreover, acidic substrates often lead to a lower CYP catalytic activity due to the disruption of the salt bridge or the hydrogen bond between the heme-propionate and the basic residue [82,83].

#### 1.2.2 H_2_O_2_-mediated epoxidation

H_2_O_2_-mediated epoxidation utilizes the hydrogen peroxide shunt bypass to achieve a highly selective C–O cyclization reaction via the coupling-II pathway. There is a demand to prevent H_2_O_2_ escape; otherwise, H_2_O_2_ will degrade the unstable Fe^III^(O_2_H_2_) intermediate into a resting state, causing ineffective catalysis. This process, accompanied by the release of H_2_O_2_, is called the uncoupling pathway [23,84]. The much lower utilization rate of H_2_O_2_ than that of O_2_ results in low efficiency of the coupling-II pathway in the hydrogen peroxide shunt bypass. Promoting the binding of free H_2_O_2_ near the active center to the heme and preventing the degradation of Fe^III^(O_2_H_2_) to release bound H_2_O_2_ are effective strategies to improve the H_2_O_2_ utilization rate [85]. Acid-alcohol pairs mediating the proton transfer process are critical for transforming Cpd 0 to Cpd I, thus significantly influencing the efficiency of the coupling-II pathway [70]. Based on the above, the conversion rate of H_2_O_2_-mediated epoxidation can be improved by (i) promoting H_2_O_2_ binding, (ii) enhancing the stability of the Fe^III^(O_2_H_2_) intermediate, and (iii) enhancing proton transfer efficiency.

Promoting the binding of H_2_O_2_ to the heme improves substrate efficiency in obtaining oxygen atoms during epoxidation and can be achieved by reconstructing the substrate pocket. Mutating small residues in the substrate pocket into amino acids with large side chains can prevent the escape of H_2_O_2_ molecules near the active site to some extent; thus, more H_2_O_2_ molecules can combine with the heme to catalyze the epoxidation of the substrate. An evident example is a series of variants in the 245th alanine of CYP152B1. Compared with the wild-type, which cannot catalyze styrene epoxidation utilizing H_2_O_2_, the A245D mutant successfully catalyzed this reaction, achieving a catalytic activity (*k*_*cat*_) of 72 min^−1^ and a catalytic efficiency (*k*_*cat*_*/K*_*M*_) of 14 M^−1^s^−1^. In contrast, the A245E mutant achieved a *k*_*cat*_ and *k*_*cat*_*/K*_*M*_ up to 280 min^−1^ and 190 M^−1^s^−1^, respectively [86]. Notably, the above substitution is usually not targeted at the gated residues for fear of hindering the substrate molecules into the active site and adjusting the binding conformation.

Enhancing Fe^III^(O_2_H_2_) stability is another effective strategy to enhance the utilization of H_2_O_2_ by CYPs. Unstable Fe^III^(O_2_H_2_) intermediates release the bound H_2_O_2_, switching the catalytic pathway from the coupling-II pathway to the uncoupling pathway, resulting in ineffective catalysis. The formation of Fe^III^(O_2_H_2_) is controlled by the positively charged residues in the acid-alcohol pairs, such as Asp251 in CYP101A1 [87]. Only when the basic residue is deprotonated its carboxylate side chain is rotated upward to form a hydrophobic salt bridge with other residues, thereby breaking the hydrogen bond between H_2_O_2_ and the water chain to facilitate the generation of the Fe^III^(O_2_H_2_) intermediate [22,88]. The stability of the Fe^III^(O_2_H_2_) intermediate can be enhanced by hydrogen bond interactions of polar residues [89]. The shorter the hydrogen bond provided by the residues, the more efficient the conversion of Fe^III^(O_2_H_2_) to Cpd I. A representative example is the methionine mutation Thr213 in CYP119. The T213M mutant formed a hydrogen bond between the sulfoxide oxygen of methionine-sulfoxide and the distal H of Fe^III^(O_2_H_2_), resulting in a 1.33- and 1.5-fold increase in the conversion rate and the ee value, respectively, when the substrate was *cis*-*b*-methyl styrene [90].

**Fig. 3:**
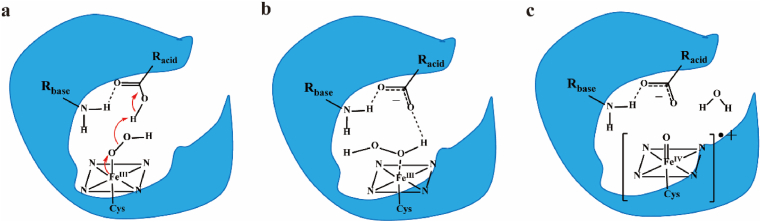
Artificial acid-alcohol pairs in the active site of CYPs **a.** Common principle of the Cpd 0 in the presence of acid-alcohol catalytic residues in CYPs; **b.** Common principle of the Fe^III^(O_2_H_2_) intermediates in the presence of acid-alcohol catalytic residues in CYPs; **c.** Common principle of the Cpd I in the presence of acid-alcohol catalytic residues in CYPs. The carboxylate group comes from a glutamic acid residue. The amino group derives from a histidine or an arginine residue, whose N–H bond stabilizes the negative charge of the acid-base pair.

In addition to controlling the formation of Fe^III^(O_2_H_2_), acid-alcohol pairs are mainly responsible for proton transfer to promote the generation of Cpd I. Artificial construction of more efficient acid-alcohol pairs can enhance proton transfer efficiency and accelerate Cpd I generation, further improving H_2_O_2_-mediated epoxidation activity [70,72]. For example, the T268E mutant of CYP102A1 acquired oxygenation activity depending on H_2_O_2_. The T213E mutant of CYP119 showed a tenfold increase in epoxidation activity compared to the wild-type [14,86]. Compared to the conserved threonine as a non-ionizing polar amino acid, glutamate carries negative charges and thus shifts the protonation/deprotonation state more flexibly, speeding up the proton transfer of the acid-alcohol pair channel (Fig. 3). In addition, dummy molecules with carboxyl and imidazolyl groups can be inserted into CYPs as alternative acid-alcohol residues, shifting the enzyme state from the resting low-spin to the active high-spin [91,92]. For instance, the addition of the dual functioning molecule N-(*ω*-imidazolyl)-hexanoyl-l-phenylalanine (Im-C6-Phe) to the F87A mutant of CYP102A1 resulted in a 30-fold improvement in the total turnover number (TON) for epoxidation [93].

### 1.3 Ketonization catalyzed by CYPs

**Fig. 4:**
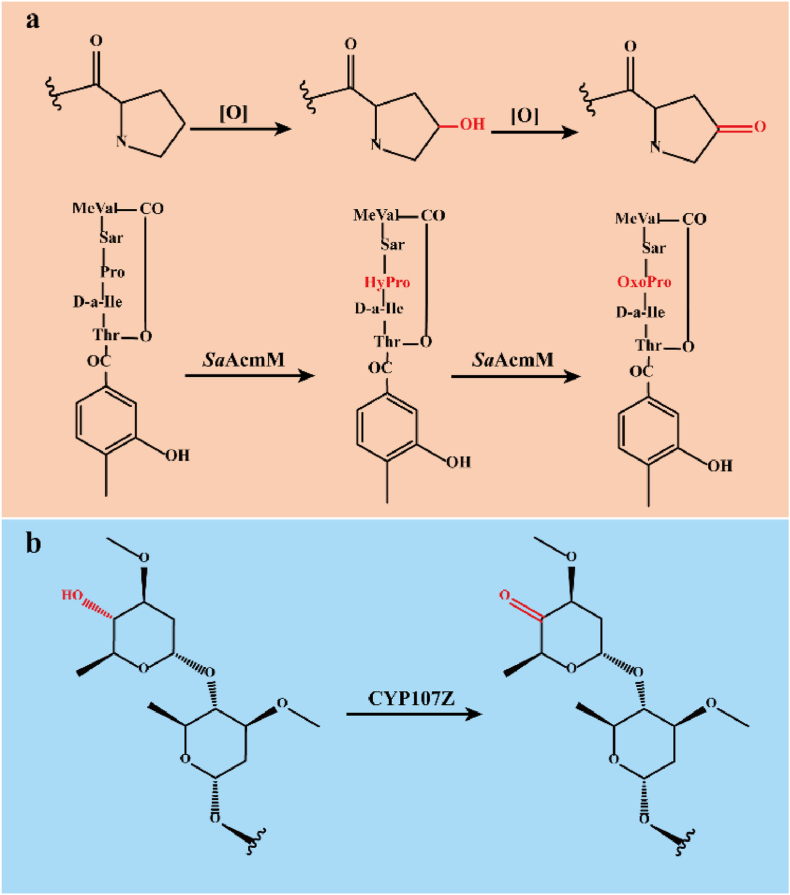
The ketonization reactions catalyzed by CYP107Z family and *Sa*AcmM **a.** The conversion of actinomycin to 4-oxoproline-actinomycin catalyzed by *Sa*AcmM. **b.** The conversion of avermectin B1 to 4″-oxo-avermectin B1 catalyzed by CYP107Z. Abbreviations: Sar, sarcosine; MeVal, *N*-methyl-l-valine.

In addition to hydroxylation and epoxidation, ketonization is also an oxidation reaction of C–H bonds catalyzed by CYPs. However, there are very few cases of ketonization; therefore, this chapter will take representative ketonization catalyzed by CYPs to introduce the ketonization mechanism. In contrast to the one-step catalysis of hydroxylation and epoxidation, the ketonization of methylene is a two-step reaction, and it is only mediated by O_2_ [18,94]. Using the classic two-electron oxygen transfer cycle, rare CYPs continuously introduce two hydroxyl groups to the same carbon atom of substrate molecules, followed by spontaneous dehydration to form ketones. First, CYPs hydroxylate substrate molecules to obtain intermediates. Subsequently, the same CYPs catalyze the C atom with a hydroxyl group in substrate molecules ketonization to obtain final ketone products. In the early stage of the reaction, two successive steps result in the conversion of all initial substrate molecules to ketones. However, the accumulation of hydroxyl by-products usually far exceeds that of ketones in the middle and late stages of the reaction [18]. Therefore, the ketonization catalyzed by CYPs is usually inefficient, and this phenomenon may be explained by the ketonization catalyzed by *Sa*AcmM (Fig. 4a).

Oxidizing a secondary alcohol group is the proven mechanism of *Sa*AcmM to catalyze ketonization, while the catalytic mechanism of other CYPs with similar functions has not been clearly elucidated. The uncoupling relationship between hydroxylation and ketonization is mainly caused by feedback inhibition of ketone products, but it has no parallel effect on the hydroxylation reaction. The spatial difference between the two reactions results in the accumulation of hydroxyl products and the formation of a small number of ketones [18]. This feedback inhibition can be relieved by separating the final products from the system in time or gene engineering to remove the final product inhibition.

So far, only a few CYPs catalyzing ketonization have been discovered, among which the most well-known are *Sa*AcmM and CYP107Z12 (Ema). *Sa*AcmM from *Streptomyces antibioticus* is a new family of peptidylproline-ketonizing CYPs and catalyzes proline hydroxylation and ketonization in the actinomycin precursor [95]. CYP107Z12 (Ema) from *Streptomyces hygroscopicus* catalyzes the conversion of avermectin B1 to its hydroxyl and ketone derivatives (Fig. 4b) [96]. Thus far, there have been few reports on ketonization catalyzed by CYPs, and related protein engineering strategies need to be further developed.

### 1.4 Applications of C–H and C=C oxygenation reactions

Among the three types of C–H and C=C oxygenation reactions catalyzed by CYPs, hydroxylation and epoxidation are more widely used in practical applications. The direct introduction of C–O bonds provides a potent method for synthesizing complex compounds and pharmaceutical intermediates, thus increasing the value of cheap raw materials [[97], [98], [99]].

#### 1.4.1 Biosynthesis with CYPs catalyzed C–H and C=C oxygenation

**Fig. 5:**
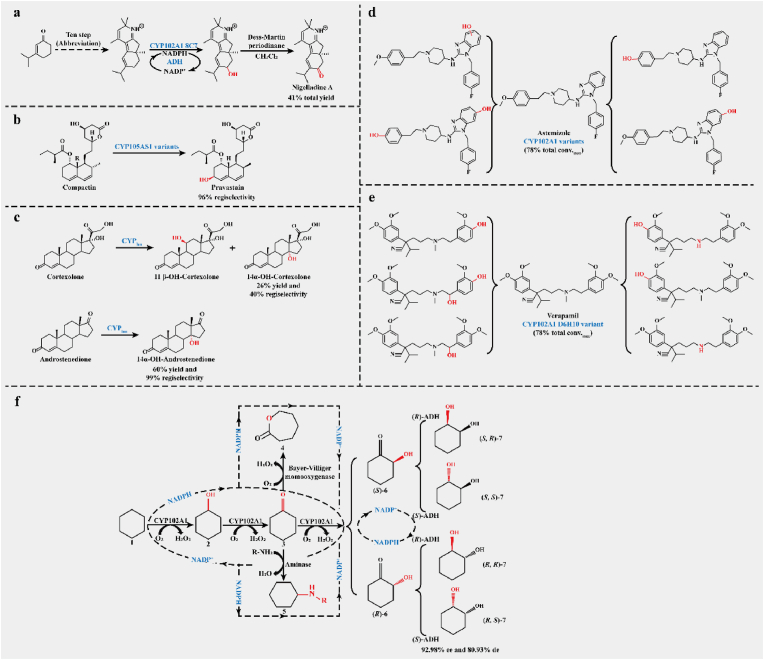
Hydroxylation reactions catalyzed by CYPs in production **a.** Starting from 3-isopropyl-2-cyclohexenone to produce nigelladine A; **b.** Synthesizing pravastatin from compactin; **c.** Introducing a hydroxyl group into androstenedione and cortexolone to produce 14*α*-OH-androstenedione and 14*α*-OH-cortexolone respectively; **d.** Converting astemizole to mammalian metabolites; **e.** Converting verapamil to mammalian metabolites; **f.** Three types of representative multi-enzyme cascades involving O_2_-mediated hydroxylation and ketonization catalyzed by CYP102A1. ADH, alcohol dehydrogenase.

As an early focus of CYP whole-cell catalysts, hydroxylation has been partially and commercially applied, especially in drug synthesis. High-selectivity hydroxylation catalyzed by CYPs plays a vital role in synthesizing antibiotics and sterol derivatives. For example, an engineered CYP102A1 mutant was applied to the enantioselective total synthesis of norditerpenoid alkaloid nigelladine A. This catalyzes highly selective hydroxylation of the allylic C–H bond in the presence of three other oxidizable sites, eventually resulting in 43% yield based on redesigned biocatalytic process (Fig. 5a) [100]. The CYP105AS1 mutants from *Amycolatopsis orientalis* synthesized pravastatin from compactin and completely reversed wild-type stereoselectivity, resulting in pravastatin to 6-epi-pravastatin ratio changing from 3:97 to 96:4 (Fig. 5b) [44,45]. CYP_lun_ from *Curvularia lunata* converted androstenedione and cortexolone to 14*α*-OH-androstenedione (60% w/w yield and regioselectivity up to 99%) and 14*α*-OH-cortexolone (26% w/w yield and regioselectivity up to 40%), respectively (Fig. 5c) [101]. During the biosynthesis of paclitaxel, half of the enzymatic reactions were catalyzed by CYPs. The CYP725A family played a significant role [102], such as the oxygenation of taxadiene catalyzed by CYP725A4 and the titer of T5*α*-ol-taxadiene reached 78 mg/L and 570 mg/L in *Saccharomyces cerevisiae* [103] and *E. coli* [104], respectively. Furthermore, various CYP102A1 mutants produced almost all mammalian metabolites of verapamil and astemizole through hydroxylation, with 78% highest total conversion rate and ≥75% selectivity (Fig. 5d–e) [105]. In fact, hydroxylation catalyzed by CYPs is usually the first step in activating the starting molecules in multi-enzyme cascades [97,99]. For example, a mature cascade consisting of a highly selective CYP102A1 variant and alcohol dehydrogenase (ADH) from *Lactobacillus kefiri* transformed cyclohexane to any stereoisomer of cyclohexane-1,2-diol, up to 92%–98% ee and 80%–93% de (Fig. 5f) [106]. Based on the CYP-ADH cascade, the complement of Bayer–Villiger monooxygenase can produce lactones, such as 2-oxocanone and ε-caprolactone [107]. Furthermore, the combination of tyrosine phenol lyase with the CYP102A1 variant catalyzed asymmetric amination starting from mono-substituted benzenes and resulting in 5.2 g/L l-DOPA surrogates [108].

**Fig. 6:**
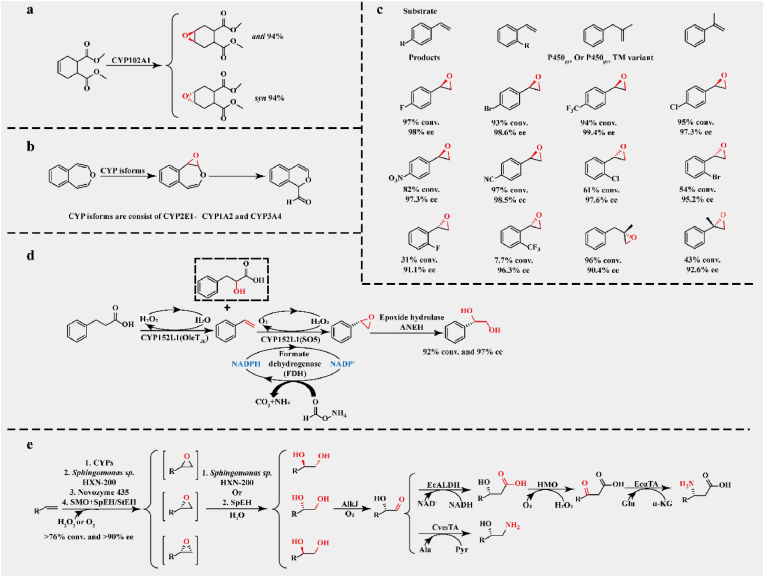
Epoxidation reactions catalyzed by CYPs in production **a.** The diastereoselective epoxidation of dimethyl *cis*-1,2,3,6-tetrahydrophthalate catalyzed by CYP102A1; **b.** Converting 4,5-benzoxepin to 2,3-epoxyoxepin via CYP isoforms; **c.** A series of epoxidation of substituted styrenes catalyzed by P450_pyr_ variant; **d.** The two-step oxidative cascade catalyzed by OleT_JE_ and CYP102A1; **e.** A general model for multi-enzyme cascades involving CYPs-catalyzed epoxidation.

Both hydroxylation and epoxidation can use alkenes and other cheap and readily available starting materials to obtain high added-value products. For instance, a series of terminal alkenes were converted to the corresponding (*S*)- or (*R*)-epoxides using engineered CYP102A1 mutants, with epoxidation turnovers up to 1370, catalytic selectivity up to 95%, and enantioselectivity up to 83% ee (Fig. 6a) [109]. In addition, CYP isoforms consisting of 2E1, 1A2, and 3A4 converted 4,5-benzoxepin to 2,3-epoxyoxepin; this is a reactive intermediate that rapidly undergoes ring-opening and isomerization to form 1*H*-2-benzopyran-1-carboxaldehyde, which is the primary method for benzene to open the ring and obtain muconaldehyde (Fig. 6b) [110]. A P450pyr triple mutant efficiently catalyzed (*R*)-selective epoxidation of *para*-substituted styrenes, resulting in 82%–97% conversion rate and 98.5%–99.5% ee (Fig. 6c) [111]. The generated (*R*)-*para*-substituted styrene oxides are important pharmaceutical intermediates that are hardly produced by other chemical or enzymatic systems. A novel P450tol from *Rhodococcus coprophilus* co-expressed with glucose dehydrogenase converted *meta*- and two *ortho*-substituted styrenes to their corresponding (*R*)- oxides, with 74%–99% conversion rate and 92%–99% ee [112]. In the multi-enzyme cascades, CYP-catalyzed epoxidation first introduces oxirane, followed by hydrolases, such as epoxidase from glycol, to the substrate molecules through ring-opening reactions, realizing the introduction of two oxygen atoms to adjacent C atoms in the substrate molecules. For instance, the biocatalytic cascade consisting of P450 OleT_JE_, CYP102A1(SO5), and epoxide hydrolase ANEH converted 3-phenyl propionic acid to (*R*)-phenyl glycol, resulting in 97% ee and a conversion rate of up to 92% (Fig. 6d) [113]. OleT_JE_ first decarboxylated the starting substrate to styrene, which was converted to (*R*)-styrene oxide by CYP102A1 (SO5) with high (*R*)-selectivity, followed by an ANEH-catalyzed ring-opening reaction to obtain (*R*)-phenyl glycol. For *ortho-* or *meta-*substituted 3-phenyl propionic acid derivatives, the above cascades usually led to a conversion rate of >76% and ee of >90%. The multi-enzyme cascade, epoxidation, and ring-opening have been widely used in various organic syntheses with high selectivities, such as the asymmetric synthesis of vicinal diols, 1,2-amino alcohols, *α*-hydroxy acids, and *α*-amino acids from alkenes (Fig. 6e) [114].

#### 1.4.2 Heterologous expression of CYPs for biosynthesis

In practical application, CYPs whole-cell biocatalyst is the major form because of the stability of active components and the cofactors provided by hosts [115]. Therefore, improving the heterologous expression of CYPs for whole-cell transformation is crucial for the biosynthesis of various products. These strategies can be divided into environmental, molecular, and gene levels.

Environmental level optimization is the selection of a heterologous host, which influences gene transcription, translation, and protein modification. *E. coli* BL21(DE3) is the most common prokaryotic host and is applied to the most bacterial CYPs [116]. In contrast, yeast is generally used to express the membrane-bound CYPs, such as *Saccharomyces cerevisiae* [117] and *Pichia pastoris* [118], *Yarrowia lipolytica* [119], *Schizosaccharomyces pombe* [120], and *Arxula adeninivorans* [121] have been used as eukaryotic hosts, and their ability to modify CYPs have been studied. For example, the expression of CYP88D6 and CYP72A154 in *S. cerevisiae* [122] and that of CYP57B3 in *P. pastoris* [123] were examined.

Co-expression with protein partners and supplementation of the prosthetic group improve the expression of CYPs at the molecular level. The representative protein partners are CPRs and GroES/GroEL. For example, PPDS from *Panax ginseng* fused to CPR-ATR1 via a GSTSSGSC polypeptide linker lead to a 4.5-fold increase in catalytic activity [124]. Unlike the CPRs used widely in yeast hosts, GroES/GroEL is generally applied to the expression of CYPs in *E. coli.* CYP79A2 from *Arabidopsis thaliana* co-expressed with the heat shock protein GroES/GroEL resulted in a 4.6-fold increase in catalytic activity for *E, Z*-phenylacetaldoxime [125]. Supplementing a moderate prosthetic group can enhance the expression of CYPs in microbial hosts, most of which lack the pathway to generate heme [126]. The addition of heme (FePPIX) or 5-aminolevulinic acid (ALA) is a common strategy and ALA has better effects than FePPIX owing to the absence of an uptake system for FePPIX in the microorganism. For example, the supplementation of 0.5 mM ALA improved the activity of CYP153A33, raising the titer of 1,12-dodecanediol produced from 0.26 mM to 0.58 mM [127]. Purified membrane human CYPs (CYP3A4, CYP21A2, and CYP17A1) were obtained at a 50–100 nmol yield via exogenous FePPIX supplementation combined with the introduction of the uptake genes [128].

Strategies at the gene level include codon optimization, *N*-terminal modification and endoplasmic reticulum (ER) engineering. Preferential codons for heterologous hosts increased the translation of inserted genes and improved the soluble expression of CYPs [129]. For example, the catalytic activity of integrated CYP176A1 for hydroxycineole was increased by 5.4-fold after optimizing codons for *E. coli* [130]. Modification of the *N*-terminal hydrophobic signal sequence is a common strategy for eukaryotic CYPs to decrease the formation of inclusion bodies [131]. This can be done by replacing the 15 *N*-terminal amino acids with the eight-residue hydrophilic “MALLLAVF” peptide from bovine in CYP71AV1 expressed by *E. coli*, leading to an 8-fold increase in the catalytic activity for dihydroartemisinic acid [132]. In addition, the “AKKTSSKGKL” peptide from rabbit, which was introduced to the *N*-terminus of AmI2′H from *Astragalus membranaceus* in *E. coli* [133], is an alternative choice. Most eukaryotic CYPs depend on inner membranes to achieve posttranslational modification, ER engineering expands the ER to provide more space for the correct folding of CYPs through the deletion or overexpression of regulatory genes. For example, the deletion of *PAF1*, which is responsible for encoding the phosphatidic acid phosphatase in *S. cerevisiae*, resulted in the obvious expansion of the ER and improved the expression of CYP716A12, CYP72A68, and CYP72A67. As a result, the titer of sapogenins *β*-amyrin, medicagenic acid and medicagenic-28-*O*-glucoside showed an 8-, 6-, and 16-fold increase, respectively [134]. Lastly, overexpression of *INO2* gene as a lipid-regulatory factor also led to the expansion of the ER and an 8-fold increase in the catalytic activity of CYP for protopanaxadiol [135].

## Summary and outlook

The C–H and C=C oxygenation processes catalyzed by CYPs provide a convenient and efficient way to insert oxygen atoms into inert molecules. CYP-catalyzed C–H and C=C oxygenation reactions are widely applied in producing chemicals and pharmaceutical intermediates to activate cheap raw materials and insert O atoms in a single catalyst or multi-enzyme cascades. Using biotechnology methods, such as protein engineering, the catalytic efficiency of hydroxylation, epoxidation, and ketonization catalyzed by CYPs can be improved, and regioselectivity and enantioselectivity regulation are enhanced. To some extent, this provides more efficient catalysts and increases the synthetic applications of C–H and C=C oxygenation, especially for the synthesis of drug intermediates with lower cost and more straightforward process. In addition, with advancements in protein engineering technology, the new functions of CYPs have been further explored, such as the formation of C–C, C–N, C–B, and C–Si bonds via amination, alkylation, and carbine transfer [136,137]. Future efforts should focus on designing better CYPs from scratch for more challenging reactions via computer simulation. C–H and C=C oxygenation reactions, including hydroxylation, epoxidation, and ketonization, and various new functions catalyzed by CYPs will significantly contribute to agriculture, drug development, food and feed additives, and other fields.

## CRediT authorship contribution statement

**Yu Yan:** Writing – original draft, Data curation. **Jing Wu:** Data curation. **Guipeng Hu:** Data curation. **Cong Gao:** Data curation. **Liang Guo:** Writing – review & editing. **Xiulai Chen:** Writing – review & editing. **Liming Liu:** Writing – review & editing. **Wei Song:** Writing – review & editing.

## Declaration of competing interest

The authors declare no conflict of interest.
